# Cavitatory mesenteric lymph node syndrome: A rare entity

**DOI:** 10.4103/0971-3026.59759

**Published:** 2010-02

**Authors:** Vibhuti, Kalia Vishal, Anakhvir Gill

**Affiliations:** Department of Radiodiagnosis, Dyanand Medical College and Hospital, Ludhiana - 141 001, India

**Keywords:** Cavitatory mesenteric lymphnode syndrome, celiac disease, CT

## Abstract

Celiac disease is a gluten sensitive enteropathy that involves an abnormal immunological response to glutens in wheat, rye etc. It predominantly involves the small intestinal mucosa, though, extra luminal manifestations can also occur. One rare extraluminal manifestation is cavitatory mesenteric lymph node syndrome. It occurs in refractory celiac disease and is associated with poor prognosis due to various complications. The diagnosis is often made on imaging when cystic mesenteric lymph nodes with fat-fluid levels are seen and this can then be confirmed by histopathological examination. We recently had a typical case where we were able to make this diagnosis.

## Introduction

Celiac disease is a genetically based gluten sensitive enteropathy in which there is inflammatory response and subsequently mucosal injury to bowel due to gluten protein (found in wheat, oat etc). The patient usually presents with diarrhea and steatorrhoea, weight loss, fatigue and abdominal pain. Extra intestinal manifestations include iron deficiency anaemia, osteopenia, apthous ulcers, dental enamel defects, peripheral neuropathy, ataxia, dementia, seizures and dermatitis herpetiformis.[1]

### Clinical details

**Figure 1:**
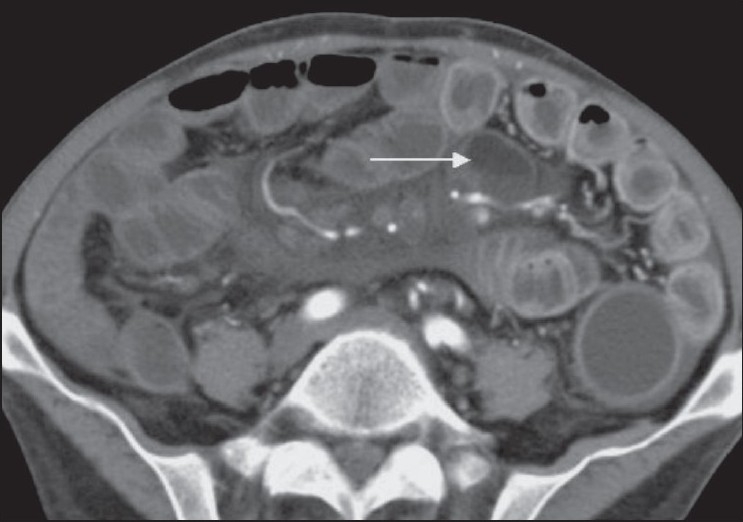
Axial CT scan shows mesenteric lymphadenopathy (arrow) with fat-fluid levels

**Figure 2:**
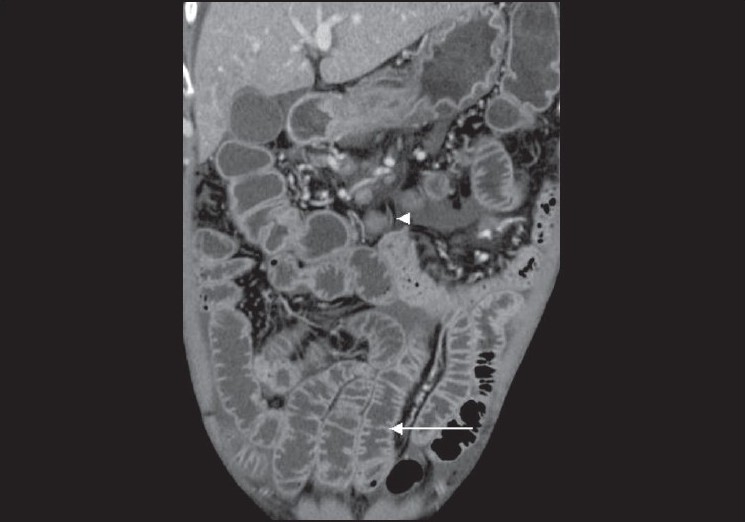
Coronal CT scan shows increased number of folds in the ileum (arrow) with mesenteric lymphadenopathy (arrow heads)

A 54 year-old male presented with a history of diarrhea, generalized weakness, and significant weight loss of three years' duration. He was diagnosed to have celiac disease in 2006 when a duodenal biopsy showed total villous atrophy with an increase in intraepithelial lymphocytes. The anti-tissue transglutamin (IgA) levels were found to be increased (12.40 E. Units). The patient had been on a gluten-free diet since then, but still occasionally had relapses. Physical examination of the patient revealed no remarkable findings. Laboratory investigations revealed microcytic hypochromic anaemia (iron deficiency anemia), elevated alkaline phosphatase, *i.e*., 534 IU/L (normal range: 64-306 IU/L) and hypoproteinemia with a serum albumin of 2.37 g/L (normal range: 3.5-5.2 g/ dL), globulins 3.77 g/dL (normal range 2.5-3.5 g/dL) and an albumin: Globulin ratio of 0.63 (normal range: 1-2.1). Ascitic fluid showed no acid-fast bacilli, no evidence of malignant cells, and normal adenosine deaminase levels. CT scan images of the abdomen and pelvis were obtained and revealed diffuse mucosal fold thickening of the jejunum and ileum with ascites, a fatty liver, and cholelithiasis. Multiple, enlarged, low-attenuating lymphnodes with fat containing areas (−15 to −45 Hounsefield units) and some with fat-fluid levels were seen in the mesentery and retroperitoneum [Figures 1 and 2]. Based on these findings, a diagnosis of cavitatory mesenteric lymph node syndrome (CMLS) with refractory celiac disease was considered. Fine needle aspiration cytology, performed from one of the enlarged lymph nodes showed the presence of milky fluid. On microscopic examination, the smear was found to be acellular with a background of thick, amorphous material with cholesterol clefts in between. No granulomas or malignant cells were seen. Ziehl-Neelsen stain for acid-fast bacilli was negative. Histopathology findings were consistent with cavitatory mesenteric lymph node syndrome (CMLS).

The patient thereafter developed abdominal distension and fever for which he was hospitalized for about 10 days. In the hospital, upper gastrointestinal endoscopy and duodenal biopsy were performed, which showed total villous atrophy, though the anti-tissue transglutamin levels were normal. The patient had tense ascites, which showed a total leukocyte count of 10,000 with 98% polymorphs. The patient was put on intravenous antibiotics and his general condition improved, following which he was discharged in a stable condition. Presently, the patient is on a gluten-free diet with nutritional supplements. No follow up scanning has been done till date.

## Discussion

Celiac disease is a genetically based, gluten-sensitive enteropathy in which there is an inflammatory response and subsequent mucosal injury to the bowel due to gluten protein (found in wheat, oats etc). The patient usually presents with diarrhea and steatorrhoea, weight loss, fatigue, and abdominal pain. Extraintestinal manifestations include iron deficiency anemia, osteopenia, apthous ulcers, dental enamel defects, peripheral neuropathy, ataxia, dementia, seizures, and dermatitis herpetiformis.[1] The diagnosis is usually based on clinical features, jejunal biopsy, response to a gluten-free diet, and elevated levels of antigliadin antibodies.

Laboratory investigations may show evidence of iron/folate deficiency anemia, elevated alkaline phosphatase, and deranged liver function tests. Hypoproteinemia, hypocalcemia, coagulopathy, and IgA antiendomysial antibodies are 85-98% sensitive and 97-100% specific. Jejunal biopsy reveals villous atrophy with hyperplastic crypts and increased lymphocytes, eosinophils, and mast cells in the lamina propria.[2]

Imaging may act as an adjunct in the diagnosis of this entity. USG is the initial modality of choice; various USG signs have been described by various authors but are nonspecific. These include moderate dilatation of a fluid-filled, flaccid small bowel, increased peristalsis, and moderate thickening of the bowel wall. The incidence of mesenteric lymphadenopathy is variable in these patients (0-12%).[3]

The indications for CT scan include patients with refractory disease or complications like ulcerative jejunoileitis, malignancy, or CMLS.[3] The spectrum of CT findings includes fewer folds per inch in the proximal jejunum and increased number of folds in the ileum, transient intussusceptions, ascites, splenic atrophy, and low-attenuating mesenteric lymph nodes. Lymph nodal enlargement is variably seen in celiac disease and can be due to reactive hyperplasia that is secondary to ulcerative jejunitis, reactive hyperplasia, CMLS, or lymphoma. Lymph nodes can be low-attenuating (also seen in Whipple's Disease).[1]

Cavitatory lymph node syndrome is a rare complication of refractory celiac disease characterized by cavitatory nodes, splenic atrophy, and villous atrophy of the small bowel mucosa. This syndrome was first described by Hemet *et al*. in 1969.[4] It is a rare complication of refractory celiac disease and is of unknown etiology and pathogenesis. Various hypotheses include hemorrhagic infarction due to chronic immune stimulation or mesenteric lymphadenopathy that is secondary to lymphatic obstruction.[4] The role of imaging in the diagnosis of the condition is pivotal. It has to be differentiated from tuberculosis and lymphoma, which may involve other lymph node groups in addition to mesenteric lymph nodes; splenomegaly is more common instead of atrophy.

It is likely to be a benign process, if CT scan shows a visible cleavage plane between the nodes and the great vessels.[5] Fat-fluid levels in the lymph nodes are specific for celiac disease and are a feature of CMLS, as seen in our case. The importance of diagnosing this entity can never be underestimated as its awareness can obviate unnecessary invasive procedures. Its presence, usually in refractory disease implies a poorer prognosis and is often associated with superadded infections especially pneumococcal as well as sepsis, cachexia, intestinal hemorrhage and electrolyte imbalance.[1,4]
